# Current Status of and Future Developments in Acute Stroke Management

**DOI:** 10.3390/jcm12134477

**Published:** 2023-07-04

**Authors:** Nikolaos Ntoulias, Alex Brehm, Marios-Nikos Psychogios

**Affiliations:** Department of Interventional and Diagnostic Neuroradiology, University Hospital Basel, 4031 Basel, Switzerland; alex.brehm@usb.ch (A.B.); marios.psychogios@usb.ch (M.-N.P.)

Stroke treatment has advanced rapidly over the last few years. Since 2015 and following the completion of five randomized clinical trials (RCTs), endovascular thrombectomy (EVT), in conjunction with intravenous thrombolysis, has become the gold standard of treatment for large vessel occlusions (LVOs) of the anterior circulation [1,2,3,4,5].

The current year was another breakthrough year for stroke care, as the results of three RCTs demonstrated the benefit of EVT even for large-core strokes. In those studies, in a heterogeneous population of patients from various continents, the use of endovascular thrombectomy within 24 h of symptom onset was found to be safe and associated with improved functional outcomes for individuals suffering from large-core ischemic strokes. Notably, the benefits of endovascular thrombectomy extend to all subgroups of patients with large cores. This finding underscores the significant treatment effect of endovascular thrombectomy, which remains robust even in the presence of predictors of poor outcomes such as a large infarct core [6,7,8].

In the case of LVOs, advanced imaging and often automatic calculation of perfusion-derived infarct core volumes and penumbral volumes have become crucial for clinical decision-making in the late stages of illness [9].

On the one hand, advancements in imaging technology and the development of new devices for mechanical thrombectomy (MT) have addressed some challenges in the field of stroke care. However, these advancements have also given rise to new questions, particularly concerning the potential patient benefit of EVT for strokes caused by medium and distal vessel occlusions (MeVOs and DVOs: e.g., DISTAL NCT05029414), with multiple RCTs actively enrolling patients as a result.

At the same time, steps are being taken toward popularizing the use of magnetic resonance (MR) imaging for stroke. New evidence suggests that low-field MR imaging can offer a feasible alternative to its expensive 1.5-T counterparts, thus making MR imaging available to a broader spectrum of healthcare systems and, subsequently, patients [10,11].

Furthermore, experts continue to debate workflow optimization, more specifically if a one-stop management approach for stroke can improve outcomes by minimizing intrahospital times, notably door-to-groin and groin-to-recanalization times. One-stop management has the advantage over direct-to-angiography approaches in that the diagnosis of an LVO is made with non-invasive flat-detector CT angiography [12].

While this is the status quo of stroke care and research, research efforts in the future will need to focus on addressing unanswered questions regarding other stroke-related pathologies, namely intracranial atherosclerosis and medium vessel occlusion strokes [13].

## Data Availability

All articles sited are openly available.
